# Microbubble–Nanoparticle Complexes for Ultrasound-Enhanced Cargo Delivery

**DOI:** 10.3390/pharmaceutics14112396

**Published:** 2022-11-07

**Authors:** Rachel Chapla, Katherine T. Huynh, Carolyn E. Schutt

**Affiliations:** 1 Cancer Early Detection Advanced Research Center, Oregon Health and Science University, Portland, OR 97201, USA; 2 Department of Biomedical Engineering, Oregon Health and Science University, Portland, OR 97239, USA

**Keywords:** ultrasound, targeted drug delivery, microbubble, cavitation, sonoporation, nanoparticle

## Abstract

Targeted delivery of therapeutics to specific tissues is critically important for reducing systemic toxicity and optimizing therapeutic efficacy, especially in the case of cytotoxic drugs. Many strategies currently exist for targeting systemically administered drugs, and ultrasound-controlled targeting is a rapidly advancing strategy for externally-stimulated drug delivery. In this non-invasive method, ultrasound waves penetrate through tissue and stimulate gas-filled microbubbles, resulting in bubble rupture and biophysical effects that power delivery of attached cargo to surrounding cells. Drug delivery capabilities from ultrasound-sensitive microbubbles are greatly expanded when nanocarrier particles are attached to the bubble surface, and cargo loading is determined by the physicochemical properties of the nanoparticles. This review serves to highlight and discuss current microbubble–nanoparticle complex component materials and designs for ultrasound-mediated drug delivery. Nanocarriers that have been complexed with microbubbles for drug delivery include lipid-based, polymeric, lipid–polymer hybrid, protein, and inorganic nanoparticles. Several schemes exist for linking nanoparticles to microbubbles for efficient nanoparticle delivery, including biotin–avidin bridging, electrostatic bonding, and covalent linkages. When compared to unstimulated delivery, ultrasound-mediated cargo delivery enables enhanced cell uptake and accumulation of cargo in target organs and can result in improved therapeutic outcomes. These ultrasound-responsive delivery complexes can also be designed to facilitate other methods of targeting, including bioactive targeting ligands and responsivity to light or magnetic fields, and multi-level targeting can enhance therapeutic efficacy. Microbubble–nanoparticle complexes present a versatile platform for controlled drug delivery via ultrasound, allowing for enhanced tissue penetration and minimally invasive therapy. Future perspectives for application of this platform are also discussed in this review.

## 1. Introduction

An important design consideration in drug delivery systems is targeting the delivery to the intended or afflicted tissue. Systemic delivery of drugs is commonly implemented because it is cost-effective, technically straightforward, and minimally invasive. However, employing this method without a targeting element commonly results in off-target effects, sub-optimal delivery to the active site, and high uptake and clearance by the immune system. Systems for directed delivery to the target tissue protect drugs from environmental degradation and decrease off-target release, reducing the necessary administered dose for effective treatment [1,2]. In decreasing off-target delivery, targeted delivery approaches also minimize adverse effects to healthy tissue, which is particularly critical in cases of cancer chemotherapy or administration of other cytotoxic therapeutics. Many methods of targeted delivery exist, including passive, active, and stimulus-activated approaches. Additionally, targeting strategies may be combined for enhanced targeting precision.

Passive targeting consists of designing the physical properties of the particle to optimize its accumulation at the active site [3]. This type of targeting is frequently used for cancer drug delivery, where upregulated angiogenic signaling in tumor tissue promotes rapid formation of immature vasculature. This tumor vasculature is more highly branched and permeable, or “leaky” than healthy vasculature. Due to the greater permeability of these vessels, nanoparticles are more capable of extravasating into tumor tissue. Further, poor lymphatic drainage in tumor tissue permits high nanoparticle accumulation and retention [4]. This combined effect is known as the enhanced permeability and retention (EPR) effect, and much effort has been made to design therapeutic vehicle size and shape for optimal extravasation through the leaky tumor vasculature in passive targeting approaches [3,5]. Passive targeting alone does not provide tissue specificity.

Alternatively, active targeting entails designing therapeutic vehicles for enhanced delivery to a particular target [3]. Functionalization of therapeutic vehicles with targeting moieties (e.g., antibodies, peptides, nucleic acids, carbohydrates) that recognize biomolecules upregulated in cancerous cells or the extracellular tumor microenvironment is a key area of development and has shown some clinical success in targeted drug delivery [3,6,7,8,9]. Other “smart” delivery vehicles dispense their cargo in response to tumor or inflammatory tissue-induced environmental stimuli, such as a decrease in pH or increase in enzymatic activity [3,5,10,11]. 

**Figure 1:**
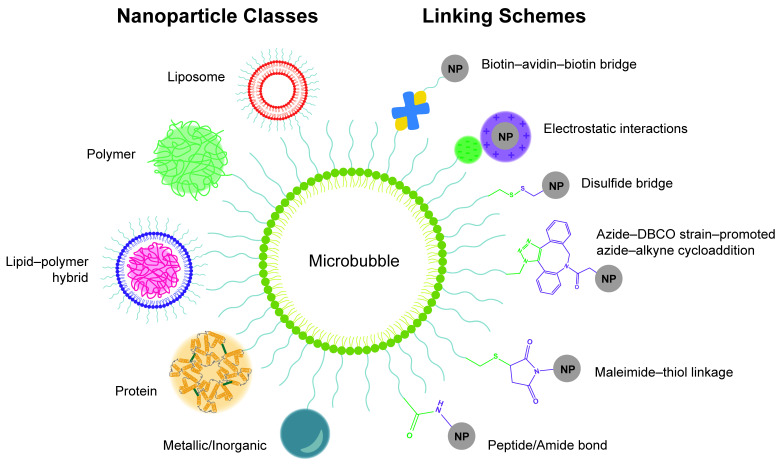
Microbubble–nanoparticle complex designs for ultrasound-controlled cargo delivery. Nanocarriers that have been linked to microbubbles include liposomes, polymer nanoparticles, lipid–polymer hybrid nanoparticles, protein nanoparticles, and metallic/inorganic nanoparticles. Schemes for conjugating nanoparticles to microbubbles include avidin–biotin bridge formation, electrostatic interactions, disulfide bridges, and multiple types of covalent bonds.

Another important method of targeted delivery that affords manual control of both location and timing of drug delivery is application of an external stimulus that triggers drug release, such as light, magnetic fields, or ultrasound [11]. Both photo- and magnetic-mediated delivery have shown utility in triggering targeted cargo delivery [12,13,14], but each present specific limitations related to tissue penetration. Focused external magnetic fields with adequate penetration and precision for guided drug delivery can be technically difficult to assemble [11], while visible and infrared light can only penetrate ≤10 mm into tissue, which is limiting in terms of clinical application for many pathologies [11,15]. In contrast, ultrasound-mediated drug delivery is particularly promising because of its technically facile and noninvasive modulation and its ability to penetrate and be focused precisely to greater depths within tissues [16]. Thus, substantial research efforts are currently focused on developing and optimizing drug delivery vehicles for ultrasound-stimulated cargo release, with a particular interest in complexing nanocarriers with ultrasound-sensitive microbubbles. Many ultrasound-controlled complexes recently introduced in this growing field have demonstrated superior targeting over unstimulated vehicles, indicating great promise for future clinical translation of this targeted therapy strategy. In response to widespread interest in the growing field, this review functions to survey the state of the art for ultrasound-responsive microbubble–nanoparticle complexes for cargo delivery. We provide an in-depth exploration of the design and synthesis of each complex component, discussing design selections, complexation and linking strategies (as summarized in Figure 1), therapeutic outcomes, and areas for future growth.

## 2. Ultrasound Stimulation

Ultrasound waves are longitudinal pressure waves of higher frequency than 20 kHz, which is above the range of human hearing [17,18,19,20,21]. These mechanical pressure waves cause oscillatory deformation of matter in the surrounding environment. Different tissues have varying resistance to ultrasound propagation, with more solid tissues reflecting more ultrasound than fluid tissues; thus, this technology has traditionally been implemented for diagnostic imaging to evaluate tissue density and morphology [22,23]. Diagnostic ultrasound imaging has had long-standing success, in large part due to its ability to deeply penetrate through tissue, allowing visualization of deep tissue morphology [24]. At frequencies around 1 MHz, ultrasound waves are minimally attenuated by tissue, allowing for significant tissue penetration [24,25]. This advantage, along with the ability to focus the ultrasound beam to small volumes within the body, has made ultrasound an attractive method of stimulation for other applications beyond diagnostics, including use for therapy and drug delivery [26,27]. The intensity of ultrasound waves may also be modulated for different therapeutic applications. High intensity ultrasound (100–10,000 W/cm^2^) causes local increases in temperature and tissue damage by thermal ablation, which can be implemented as cancer therapy, but may cause undesired damage in many applications [28,29,30]. Lower intensity ultrasound (e.g., the range of 0.125–3 W/cm^2^) is capable of inducing mechanical effects on local tissues and microparticles without causing temperature spiking [28]. Thus, researchers have harnessed this form of energy for local, on-demand drug delivery by designing micromaterials and complexes to release drugs in response to ultrasound stimulation.

### Ultrasound Stimulation of Microbubbles 

**Figure 2:**
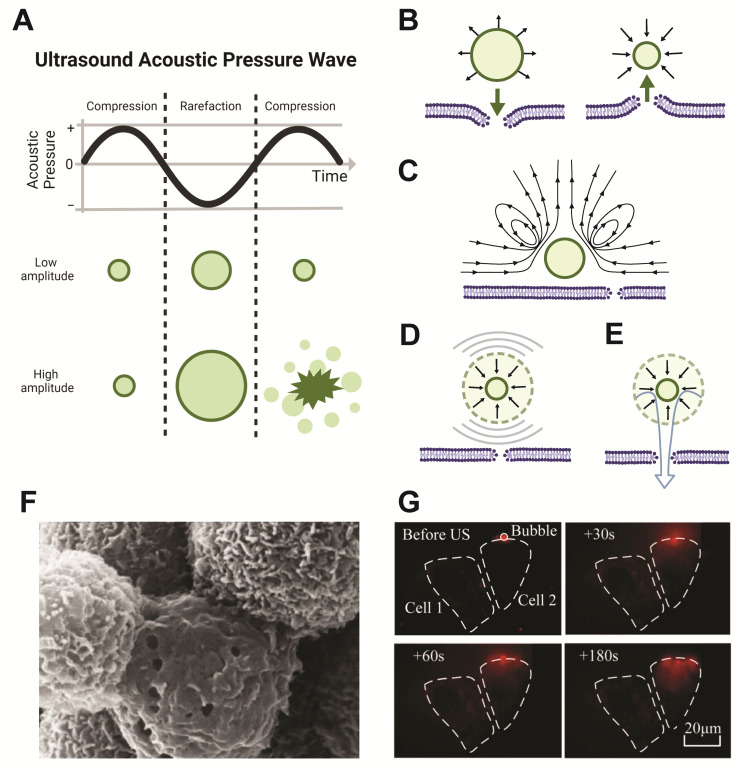
Microbubble cavitation induces sonoporation of local cell membranes. (**A**) During the sinusoidal alternating cycle of compression and rarefaction from the ultrasound pressure waves, gas-core microbubbles contract and expand in diameter. In stable cavitation (top row), occurring at lower acoustic pressure amplitudes, microbubbles stably oscillate between expansion and contraction, whereas at higher acoustic pressures, inertial cavitation can occur (bottom row), in which the microbubble expands and then implodes, collapsing and fragmenting the bubble. Schematic illustrations in (**A**) created with Biorender.com, and based on those from [57], Springer Nature, 2015, and [58], IvySpring International Publisher, 2012. (**B**–**E**) Both stable and inertial cavitation can create local dynamics which may cause sonoporation. Illustrations are based on those from [42], copyright 2017. Reproduced by permission of Taylor and Francis Group, LLC, a division of Informa plc. (**B**) Stable microbubble cavitation may cause sonoporation by pushing the cell membrane during expansion or pulling the membrane during contraction. (**C**) Stable cavitation creates local fluid microstreaming, which can also cause membrane permeation. (**D**) During inertial cavitation, resultant shockwaves from bubble implosion can create pores in cell membranes. (**E**) Bubble collapse during inertial cavitation can also cause fluid jet formation which can permeate the cell membrane. (**F**) Scanning electron microscopy images show the presence of pores in cell membranes following ultrasound exposure in the presence of microbubbles. Image reprinted from [46], copyright 2005, with permission from Elsevier. (**G**) Ultrasound stimulation in the presence of adjacent microbubbles causes cell membrane permeability, facilitating rapid cell uptake of local fluorescence marker propidium iodide (red) through pores created from sonoporation. Images reprinted from [53], Springer Nature, 2018.

Gas-core microbubble particles are highly mechanically responsive to the oscillating ultrasound pressure waves. Due to the compressibility of the gas, these microbubbles undergo volumetric oscillations, shrinking and expanding with the compression and rarefaction phases of the ultrasound pulse [31,32,33,34,35]. This oscillation is known as cavitation [36]. Two types of cavitation dynamics can occur in response to ultrasound stimulation: stable cavitation and inertial cavitation (both depicted in Figure 2A). Microbubble oscillation also results in strong backscatter of the ultrasound waves, which has enabled their use as highly effective contrast agents for ultrasound imaging [37]. 

In the process of stable cavitation, which occurs at lower ultrasound intensities, microbubbles oscillate in diameter about their equilibrium size at the frequency of the applied ultrasound wave [38]. At higher acoustic pressures, inertial cavitation can occur, where the microbubble expands to a large enough size that, upon the next compression phase, a rapid inward rush of fluid is directed toward the center of the microbubble. This causes a forceful implosion which collapses the bubble and can free attached or encapsulated cargo. The oscillation response of the microbubble is greatest at its resonant frequency, which is determined by the physical properties of the bubble itself (e.g., size, composition, coating) [30,39]. The collapse dynamics can be influenced by proximity to a surface or to other bubbles undergoing cavitation [40]. Both stable and inertial microbubble cavitation can cause a temporary increase in permeability of nearby cell membranes known as sonoporation (Figure 2B).

As microbubbles undergo stable cavitation, their rapid expansion and contraction can have a push and pull effect on adjacent cell membranes (Figure 2B) and can also generate fluid flow, known as microstreaming (Figure 2C) [41,42]. Microstreaming exerts shear stresses upon nearby cell membranes, causing transient opening of membrane pores, e.g., sonoporation [43,44,45]. During inertial cavitation, the forceful collapse of the microbubble can generate radial shock waves (Figure 2D), as well as directed fluid jets (Figure 2E), caused by the asymmetric collapse and involution of the bubble, which can disrupt nearby cell membranes [44,46,47,48,49]. Several studies have observed sonoporation-induced cellular pore sizes ranging from approximately 10 nm to multiple micrometers in diameter, using scanning electron microscopy (SEM) and atomic force microscopy (AFM) (Figure 2F) [46,47,48]. Following the formation of transient pores, evidence of pore resealing, facilitated by lysosome-mediated exocytosis [50] and extracellular calcium ions (Ca^2+^), has been observed [51]. The pore size generated is dependent upon ultrasound parameters, such as duration, number and localization of ultrasound pulses, bubble–cell proximity, and geometrical configuration of the microbubbles within the local 3D environment [52]. Upon cavitation-induced pore formation, local extracellular species may enter cells directly through these pores (Figure 2G) [53]. As such, sonoporation caused by ultrasound-stimulated microbubble cavitation is thought to play a key role in internalization of delivered therapeutics, as it creates the most direct route of delivery to cells. Thus, ultrasound-stimulated sonoporation may be leveraged to overcome challenges in targeted gene or therapeutic cargo transfer into cells [41]. 

In addition to promoting cargo delivery across cell membranes, focused ultrasound is an instrumental method for delivering cargo across the blood–brain barrier (BBB). The BBB is a neuroprotective barrier of endothelial cells joined by tight junctions impermissible to molecules larger than 400 Da [54,55]. Focused ultrasound-induced microbubble cavitation temporarily disrupts the tight junctions of the BBB without causing thermoablation of these cells, providing localized direct delivery of cargo (as large as 2000 kDa molecular weight) to brain tissue at the targeted site [55,56]. Focused ultrasound delivery is considered safer and more precise than other methods of crossing the BBB, including invasive surgeries and systemically-administered chemically-modified cargo [54,56].

Focused ultrasound-stimulated microbubble cavitation for release of therapeutic cargo is an attractive biocompatible approach for therapeutic delivery due to the deep tissue penetration and precise, noninvasive spatiotemporal control of the ultrasound stimulus. The capability to focus the acoustic energy to small mm^3^-scale volumes within deep tissue also makes ultrasound an attractive stimulus to activate drug delivery vehicles. As briefly mentioned above, microbubble cavitation behavior is largely dictated by bubble physical properties. Several considerations for design of ultrasound-responsive microbubble delivery vehicles are shell composition, microbubble size, type of gas in the core, surface functionalization, and cargo loading. Below we discuss these design parameters for different delivery applications.

## 3. Ultrasound-Responsive Microbubble Design Parameters

Microbubbles have been designed for different therapeutic applications, including the delivery of genes or other drugs, due to their unique cavitation behavior. The general microbubble design consists of an outer protective layer, or shell, and a hydrophobic gas core. The types of protective layers include phospholipids, that create a monolayer membrane, or layers composed of protein (e.g., albumin) or polymer [59]. The microbubble monolayer can be comprised of many different lipid types and mixtures and commonly consists of a phosphatidyl-choline, such as 1,2-dipalmitoyl-sn-glycero-3-phospho-choline (DPPC) [60,61,62,63], or 1,2-distearoyl-sn-glycero-3-phosphocholine (DSPC) [64,65,66,67]. These phospholipids self-arrange into monolayers across the gas/water interface with their hydrophobic lipid tails facing inwards toward the gas-filled core and hydrophilic heads facing outward toward the aqueous environment. To increase microbubble stability, polyethylene glycol (PEG) functionalized, or (PEG)ylated, lipids, such as PEG stearate [64], or 1,2-distearoyl-sn-glycero-3-phosphoethanolamine-N-carboxy(polyethylene glycol) (DSPE-PEG2000), can be incorporated. These hydrophilic PEG brush layers inhibit bubble coalescence and also increase the particle half-life in circulation by shielding the bubble from immune cell recognition [68]. The incorporation of cholesterol can modify the packing of phospholipids in the microbubble monolayer, which affects the physical properties of the lipid monolayer and the microbubble’s interaction with ultrasound [69].

Gases implemented for the microbubble core are generally inert and hydrophobic in nature to reduce dissolution into the surrounding liquid, thereby increasing microbubble stability [70,71]. Different types of gases that can be incorporated inside the phospholipid shell include: octafluoropropane [72], perfluorobutane [60], perfluorohexane [73,74] sulfur hexafluoride [75], and nitrogen [76]. Other gases may also be incorporated for different therapeutic strategies, such as oxygen to improve treatment of tumors [77,78].

In order to synthesize lipid coated microbubbles, the phospholipid mixture and gas components are combined under conditions that favor micelle self-assembly, including probe sonication, microfluidics, and high shear emulsification [79,80,81,82,83]. Probe sonication introduces energy into a lipid solution that breaks up lipid micelles while simultaneously pulling gas from above the liquid surface into the solution, forming microbubbles that are then coated by the free lipids before they reform micelles. This results in the formation of a monolayer of the lipid coating and stabilizes the gas core. Standard bulk production methods utilize ultrasonic emulsification to synthesize high volumes of microbubbles, but result in a broad size distribution [84]. Conversely, microfluidic production methods provide tighter size distributions, but operate at a much lower production rate [85]. Hybrid systems of these methods that achieve high production rate and more uniform size distributions may provide a novel fabrication platform for higher yield production [86]. The size distribution of the microbubbles is important because the microbubble resonance frequency is dependent on the bubble size [49], with one to ten microns in diameter effectively oscillating with ultrasound frequencies in the low MHz [87].

While lipid-based microbubbles have been well-established for drug and gene delivery applications, the capacity of the monolayer shell for housing cargo is low. Though less common, other macromolecules may be used to form the microbubble shell. Polymer-based microbubbles are advantageous for drug delivery due to their ability to form thicker crosslinked layers which enhance bubble stability. These polymers can achieve thicknesses of 50–150 nm in comparison to the 3–5 nm thickness of phospholipid-based microbubbles [88]. The thicker shell provides a larger space for loading therapeutics, which are entrapped within the polymer network [89]. However, the thicker membranes can be stiffer than lipid monolayers, which affects their echogenicity, or responsiveness to ultrasound stimulation, and is an important consideration for delivery applications. Successful delivery of molecular payloads across the BBB to brain tumors has been achieved using poly(n-butyl cyanoacrylate) (PBCA)-based polymer microbubbles incorporating macromolecular FITC-dextran in the shell [88]. Polymer–surfactant shell hybrids, comprised of multiple shell layers, also provide greater cargo loading capabilities and have been employed for different applications, such as dual drug release or protection of DNA cargo from enzymatic degradation [90,91,92]. Protein coated microbubbles have also been employed for gene and drug delivery purposes [93]. Proteins can be covalently crosslinked via disulfide bonds to form and stabilize a microbubble shell [94]. This design has been used to effectively deliver both adenoviruses in a rat model [95] and plasmids in vitro [93] in response to ultrasound stimulation. 

### Microbubble Customization and Cargo Capabilities

Microbubbles are promising carrier vehicles for gene or other drug delivery, enabling spatiotemporal control over delivery through the application of a focused ultrasound trigger. Customization of the microbubble structure is critical for loading different cargos intended for a wide range of different applications. 

Linking payloads to the microbubble surface can be achieved through functionalization of microbubbles with crosslinking groups or by imparting electrostatic charges on the surface. Microbubble surface charge can be modified by incorporating cationic or anionic lipids into the monolayer. This enables complexation with oppositely-charged species via electrostatic interactions. The cationic lipids 1,2-Dioleoyl-3-trimethylammonium propane (DOTAP) and N-[1-(2,3-distearoyloxy)propyl]-N,N,N-trimethylammonium methylsulfate (DSTAP) are commonly used for conferring positive charge to microbubbles, allowing negatively charged nucleic acid cargo, such as cDNA, siRNA, or mRNA, to attach to the microbubble surface [60,96,97]. Attaching nucleic acids to microbubbles protects them from enzymatic degradation in circulation [98]. DNA cargo loaded onto the microbubble surface can be verified by labeling with SYBR-gold dye and quantification of the fluorescence signal [99]. 

Microbubble surfaces can also be chemically functionalized for the attachment of specific therapeutic cargo types. For example, PD-L1 therapeutic antibodies were conjugated to PEG-coated, N-Hydroxysuccinimide (NHS)-functionalized microbubbles using a covalent amine–NHS linkage, forming an antibody–microbubble conjugate for cancer immunotherapy applications. This delivery vehicle provided protection for the therapeutic antibodies from immune recognition due to the microbubble PEG brush layer and partial blockage of the antibody Fc region by the microbubble conjugation. This design enabled effective targeted release of the antibody by focused ultrasound [100]. 

Beyond modifying microbubble membranes for cargo loading, functional groups may also be used to add targeting capabilities. Microbubbles can be targeted for specific cell types. For instance, a phospholipid microbubble conjugated (via biotin–actin bridging) with anti-CD4 was successfully targeted to CD4 positive lymphocytes that carried the microbubbles to specific regions within the body [101]. Attaching multiple ligands on the same microbubble may allow for increased specificity to cellular targets, such as to regions of atherosclerosis through conjugation of anti-VCAM-1 and anti-ICAM-1 antibodies and synthetic polymeric sialyl Lewis X [102]. Microbubbles can also be modified to leverage multiple modalities, combining imaging with drug release capabilities. For example, incorporating iron oxide nanoparticles with microbubbles allows for dual MRI and ultrasound imaging and also allows ultrasound-mediated delivery of doxorubicin [92]. 

Current methods of cargo-loading directly onto microbubbles have shown success in delivery, and increasing cargo capacity further can potentially improve therapeutic outcomes. The addition of cargo-carrying nanoparticles onto the microbubble surface is a promising area of research for increasing the loading capacity of microbubbles and expanding the types of payload that can be carried. For example, hydrophilic drugs are difficult to load directly into the microbubble lipid monolayer, but can be carried inside liposome or polymeric nanoparticles, which can be easily attached to the microbubble surface. These attached nanoparticles also increase the loading capacity beyond what can be achieved by attaching the payload directly to the limited surface area of the microbubble itself. Here we detail the range of microbubble–nanoparticle complexes that have been designed to deliver cargo in response to ultrasound stimulation.

## 4. Nanoparticle Carriers and Microbubble–Nanoparticle Complexes

Combining microbubbles with specialized carriers for cargo retention and controlled delivery provides greater molecule-loading capabilities than microbubbles, alone and expands the library of cargo types deliverable by ultrasound stimulation. Cargo vehicles can confer, or enhance, solubility to hydrophobic or amphiphilic drugs, improving biodistribution and delivery efficiency [103]. These particles improve drug safety and efficacy by preventing off-target payload release and protecting therapeutic cargo from harsh environments that may denature the molecules and diminish their therapeutic function [103,104]. Physical and chemical characteristics of carrier particles determine the class of cargo and mode of delivery, and adding these particles to ultrasound-responsive microbubbles can provide additional layers of targeting for greater precision of delivery [105]. 

The size of cargo-carrying delivery particles has been proven critical to both their function and biodistribution. Nanoparticles, in particular, have been widely employed in drug delivery due to the advantages of their size. Particles between 10–200 nm in diameter show minimal renal clearance and immune clearance in vivo [106], resulting in effective passive targeting of tumor tissue via the EPR effect [103]. To further evade opsonization and phagocytic clearance, nanoparticles may be surface-modified with a stealth layer, such as a PEG brush coating. This effectively increases circulation time as required for enhanced accumulation at the tumor site via the EPR effect [107]. Additionally, due to their size, nanoscale carriers may be internalized by cells via endocytosis. Particles between 10 and 60 nm present the optimal size for cell uptake in vitro based on size alone, and surface modifications may enhance uptake as well [106,108]. Nanoparticles have also been designed to undergo endosomal escape upon cellular uptake [109,110,111]. Finally, the high aspect ratio (surface to volume ratio) of nanoparticles can be advantageous for solubility, cargo loading, and contact-mediated interactions with cells [112,113].

Combining nanocarriers with microbubbles confers many of the unique advantages of nanoparticles to ultrasound-responsive carriers. In early systems, microbubbles and nanoparticles were co-administered or delivered to tissues in succession, showing some success in improved delivery [114,115]. For efficient microbubble cavitation-stimulated delivery directly to cells, however, the bubbles must be in close proximity to nanoparticles [116]. Accordingly, microbubble–nanoparticle delivery systems have demonstrated greater delivery efficacy when the two components are linked together [117,118,119]. Thus, in recent years, many strategies have been employed for physically and chemically linking nanoparticles to microbubbles.

A variety of ultrasound-responsive microbubble–nanoparticle complex formulations exist for different cargo delivery applications, with many recent developments and innovations in design and therapeutic application. Thus, we below describe and discuss the different existing designs for these linked microbubble–nanoparticle complexes. 

First, the type of the nanoparticle component has a critical influence on the cargo and delivery capabilities. As such, we have divided types of microbubble–nanoparticle complexes by nanoparticle class, which are outlined in Table 1. Second, several schemes exist by which the microbubbles and nanoparticles are connected, which vary in design and strength; thus, we then discuss the linking schema and how they affect the complex formation.

### 4.1. Nanoparticle Classes for Microbubble–Nanoparticle Complexes

#### 4.1.1. Microbubble–Liposome Complexes

##### Liposomes as Nanocarriers

One nanocarrier class commonly complexed with microbubbles is liposomes. Liposomes, depicted in Table 1, are vesicles composed of phospholipids assembled into an amphiphilic bi-layered membrane encircling an aqueous interior, which can hold hydrophilic cargo [141,142]. Loading molecules into liposomes for delivery provides protection to the cargo from the biological environment, reduces off-target tissue exposure to the payload, increases drug circulation time, and facilitates passive delivery via EPR [143]. 

The organization of the molecules comprising liposomes is uniquely beneficial for cargo delivery. Their double-layered phospholipid membrane is similar in structure and curvature to biological membranes, rendering them highly biocompatible and capable of direct interaction with cells. Upon contact with the cell membrane, liposomes have been shown to fuse with cellular plasma membranes for direct delivery of liposomal cargo into the cell [120,121,122]. Liposome membrane structure also enables simultaneous incorporation of multiple classes of cargo with different properties. In addition to housing cargo within its hydrophilic interior, hydrophobic cargo may also be contained in the hydrophobic compartment between lipid layers of the membrane [123]. Delivering multiple classes of therapeutics in combination is a promising approach for treatment of therapeutic-resistant cancers. Furthermore, strategies for loading cargo into liposomes are technically straightforward. 

Liposomes may be modified and functionalized [142,144]; while typically phospholipid- and cholesterol-based [141], liposomes may vary widely in design for different applications. Unilamellar liposomes for drug delivery can vary in size from ~100 nm to 800 nm in diameter, and are typically designed to be around 100 nm for optimal EPR extravasation from tumor vasculature [122,144]. Their surface functionalization can be tuned to escape immune recognition, attach charged cargo, release drug in response to a stimulus, or actively target biological molecules, which may also promote cellular uptake [122,142]. 

Liposomes provide excellent delivery of different types of cargo, but do not have effective echogenicity alone, due to their fluid-filled structure, and are more capable of US-stimulated delivery when coupled to highly echogenic microbubbles [122,125]. Linking liposomes with microbubbles confers many of the design advantages of liposomes to ultrasound-responsive microbubbles. 

##### Microbubble–Liposome Complexes for Cargo Delivery

**Figure 3:**
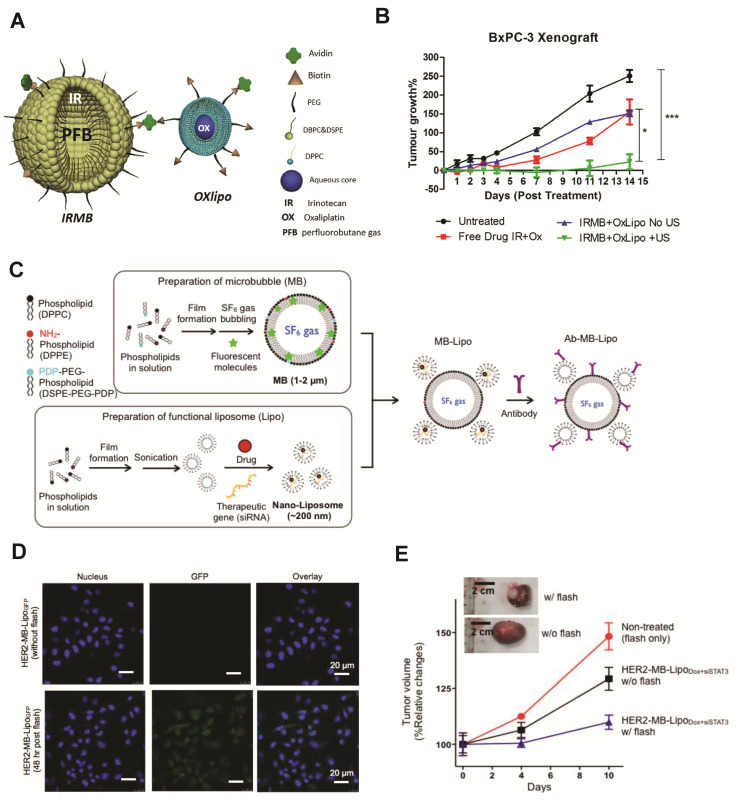
Microbubble–liposome complexes expand the types of cargo that can be delivered by ultrasound-induced microbubble cavitation, leading to improved therapeutic efficacy. (**A**) Microbubbles loaded with hydrophobic chemotherapeutic Irinotecan and liposomes loaded with hydrophilic chemotherapeutic Oxaliplatin were linked together by biotin–avidin bridging for a multi-drug cancer therapy approach. (**B**) Implementation of this system (IRMB + OxLipo + US, green) in vivo resulted in significantly lower tumor growth than delivery without ultrasound (blue), * *p* < 0.05, *** *p* < 0.0001. (**A**,**B**) reprinted from [154], copyright 2020, with permission from Elsevier. (**C**). Both DOX and therapeutic siRNA were loaded into liposomes, which were covalently conjugated to microbubbles and labeled with antibodies for multidrug active targeting. (**D**) Ultrasound-stimulated delivery (flash) of this complex promoted cell uptake and gene transfection (bottom row). (**E**) This therapeutic microcarrier complex design inhibited tumor growth when stimulated with ultrasound (flash) in vivo (shown in blue). (**C**–**E**) reprinted from [155], copyright 2014, Ivyspring International Publisher.

Many microbubble–liposome complex designs exist. Liposomal cargo housing capabilities expand the range of therapeutics that can be delivered by microbubbles, as evidenced by the studies outlined in Figure 3. As both lipid microbubbles and liposomes contain outer phospholipid layers, they are typically linked together by incorporating lipids functionalized with complementary moieties into their shells. This allows for a variety of conjugation strategies [145].

Early microbubble–liposome complexes were characterized by Kheirolomoom et al., who evaluated binding strategies and liposome parameters to maximize liposome loading efficiency onto each decafluorobutane-filled microbubble [122]. Kheirolomoom additionally evaluated liposome formulation and size for stability and complexation with microbubbles, with 5% biotinylation of PEG surface chains and 100 nm liposome diameter were found to provide efficient stability and microbubble binding. The liposome membranes were comprised of PEGylated lipids, biotinylated + PEGylated lipids, and 22-(*N*-(7-Nitrobenz-2-Oxa-1,3-Diazol-4-yl)Amino)-23,24-Bisnor-5-Cholen-3β-Ol (NBD) fluorescent cholesterol. These seminal microbubble–liposome complexes were shown to oscillate in response to ultrasound insonation, and bound liposomes were released upon ultrasound-mediated bubble oscillation or disruption [122]. Finally, ultrasound-controlled liposome delivery to PC-3 prostate cancer cells was evaluated in vitro. NBD was internalized by cells when delivered from the microbubble–liposome complex by ultrasound stimulation, but no effective transfer into cells was demonstrated by liposomes alone, microbubbles alone, or complexes without ultrasound treatment [122]. This study established the effectiveness of microbubble–liposome complexes for targeted delivery applications.

As targeting is critical for safe cancer therapies, microbubble–liposome complexes have been evaluated for chemotherapeutic delivery. Liposomes loaded with hydrophobic mitotic inhibitor paclitaxel (PTX) were conjugated to perfluoropropane-filled microbubbles via biotin–avidin linking. This early study evaluated cell uptake of cargo as a function of ultrasound parameters, finding that higher intensity and longer stimulation times increased uptake. Administration of this complex under ultrasound stimulus resulted in significantly reduced cell viability of 4T1 breast tumor cells in vitro in comparison to PTX-liposomes alone under identical ultrasound parameters. In a 4T1 breast carcinoma flank mouse model, micro-complex PTX delivery via ultrasound significantly inhibited tumor growth and showed high PTX accumulation in the tumor and low accumulation in the liver, indicating effective targeted treatment [146]. The same group later implemented this complex to deliver cancer therapeutic molecule doxorubicin (DOX) to DOX-resistant MCF-7/ADR human breast cancer cells in vitro, ameliorating therapeutic resistance more effectively than verapamil, a drug currently used to reverse multidrug resistance. DOX delivery from the microbubble–liposome complex under ultrasound stimulation resulted in the highest cellular DOX uptake, lowest efflux from cells over time, and largest decrease in cell viability compared to delivery by complexes without ultrasound, liposomes with ultrasound, and liposomes plus free verapamil with ultrasound. The higher DOX delivery corresponded to high intercellular reactive oxygen species (ROS) production, greater γ-H2AX expression, and more apoptosis [147]. Another class of therapeutics requiring targeted delivery is that of platinum-based drugs, which are effective in cancer treatment, but are toxic and highly reactive and can cause damaging off-target effects. In one complex, iproplatin was loaded into the aqueous cores of azide-tagged liposomes, which were then covalently linked to DBCO-functionalized sulfur hexafluoride-filled microbubbles. Passive drug leakage from liposomes was evaluated, finding 19% iproplatin remaining within the liposomes after six days in solution [148]. When the complexes were administered to MCF7 breast cancer cells in vitro, ultrasound stimulation enhanced cell uptake of iproplatin from the microbubble–liposome constructs, while cell uptake of iproplatin from liposomes alone with applied sonication was negligible [148]. In another covalently-linked complex, DOX-loaded maleimide-functionalized liposomes were combined with a mixture of lipids, some of which presented thiol groups. Upon mixing with hydrophobic gas, the lipids self-assembled into liposome-loaded microbubbles. This covalent maleimide–thiol linkage withstood buffer and plasma conditions, with liposomes remaining bonded to microbubbles in the absence of ultrasound stimulation. When activated by ultrasound in vitro, these complexes showed highly effective cytotoxicity to melanoma cells, with roughly the same cell viability at 5.4 µg/mL as DOX-loaded liposomes alone showed at 27 µg/µL (~60% viability). Delivery of free DOX at 0.5 µg/mL resulted in ~90% cell viability, while delivery of 0.5 µg/mL delivered from the carrier complex by ultrasound resulted in ~65% cell viability. These findings suggest that ultrasound-controlled delivery can enhance therapeutic efficiency and allow for lower administered doses of harsh therapeutic molecules, increasing overall safety [149]. In all the above studies, complexing liposomes with microbubbles and stimulating with ultrasound resulted in microbubble cavitation-induced enhancement of cell uptake and therapeutic efficacy of chemotherapeutic small molecules.

Due to their ability to protect fragile cargo in circulation, microbubble–liposome complexes have also been implemented in gene delivery. Therapeutic plasmids can be effective in upregulating pro-healing pathways and repressing pro-tumor or pathological marker expression in many disease states, but they are highly sensitive to degradation from free nucleases in circulation and have demonstrated poor accumulation at the target site due to denaturation [150]. Viral delivery vectors are effective carriers, but they can cause immune reactions and mutagenesis [151,152]. Liposomal delivery serves as a safer alternative for delivery of therapeutic nucleic acids.

Nucleic acid delivery from microbubble–liposome complexes has shown particularly promising results in treatments for liver fibrosis, for which microbubble–cationic–liposome complexes have been developed for electrostatic linkage of negatively-charged genetic material to the liposome surfaces. In one study of hepatocyte growth factor (HGF) gene delivery as a therapy for liver fibrosis, pCDH-HGF plasmid was loaded onto the cationic liposome surface of microbubble–liposome complexes. Liposome charge was imparted by cationic cholesterol DC-chol incorporation in the lipid layer, and biotin–avidin–biotin bridges linked the liposomes to the microbubbles. These complexes were administered intravenously in a rat bile duct ligation model of liver fibrosis, and ultrasound stimulation was locally applied. Enhanced echo signals from ultrasound imaging confirmed microbubble–liposome complex accumulation at the target site. This therapy reversed the fibrotic effects of the ligation as measured by local hydroxyproline content [151]. A second study targeted another fibrosis-related gene, connective tissue growth factor (CTGF). Delivery of silencing miCTGF RNA by cationic microbubble–liposome complexes downregulated CTGF gene expression in HSC-T6 cells in vitro and significantly ameliorated fibrotic factors CTGF, TGFβ1, collagen I, and α-SMA expression in the rat liver in a fibrosis model in vivo. This therapy method effectively inhibited fibrotic progression and prevented development into cirrhosis [153]. Effective gene delivery by microbubble–liposome complexes in vivo shows promise for targeted genetic therapy. 

Liposomes can support the delivery of multiple classes of cargo at once, thus, facilitating multidrug therapy for improved therapeutic outcomes. Demonstrating the expanded cargo loading capabilities of the microbubble–liposome complex, one study delivered a known synergistic combination of hydrophilic and hydrophobic drugs to a BxPC-3 human pancreatic tumor model in severe combined immunodeficient (SCID) mice. Combinatorial therapies implemented in pancreatic cancer treatment often cause off-target toxicity and adverse systemic effects, presenting a need for improved targeted therapies. In this design, hydrophobic drug irinotecan was incorporated into the lipid membrane layer of a perfluorobutane-filled microbubble, while hydrophilic oxaliplatin was loaded into the aqueous liposome core, and the two carriers were linked together via the biotin–avidin–biotin bridge (Schematic shown in Figure 3A). In vivo delivery of this complex under ultrasound exposure significantly impeded tumor growth in vivo without causing systemic toxicity, resulting in tumors 136% smaller than those treated with the complex without ultrasound and those treated with free multidrug therapy (Figure 3B). These results underscore the importance of both the microcarrier complex and the ultrasound stimulation in effective delivery for efficacious treatment of drug-resistant pancreatic tumors [154]. In another multi-drug delivery study for breast cancer therapy, DOX was dissolved in the aqueous liposome core and therapeutic RNA was incorporated in the lipid layers by including a plasmid–protamine complex in the lipid film during liposome synthesis. A green fluorescent protein (GFP) tester plasmid was first implemented to evaluate cell transfection and then was replaced by therapeutic siRNA siSTAT3 (signal transducer and activator of transcription 3, cell proliferation-promoting transcription factor). Liposomes were conjugated to microbubbles via a Thiol–pyridyldithiopropionate (PDP) disulfide bond and surface labeled with anti-HER2 antibodies for targeting HER2+ breast cancer cells (Figure 3C). Upon ultrasound-stimulated delivery to SkBr3 (breast cancer cells over-expressing HER2) in vitro, cells expressed GFP (while there was no expression when microcarriers were delivered without ultrasound) (Figure 3D). In the therapeutic model, ultrasound exposure enhanced cell internalization of DOX. When complexes loaded with both DOX and siSTAT3 were exposed to ultrasound, the treatment significantly decreased expression of pro-tumor and pro-mitotic factors STAT3, Cyclin D1, and c-Myc, reduced cell viability, and inhibited tumor growth (Figure 3E), indicating efficacy for cancer therapy [155].

Finally, one unique model conferred ultrasound responsiveness to liposomes not by linking the particle surfaces together, but by loading microbubbles within a large, micron-scale liposome aqueous core, along with the therapeutic cargo. Cavitation of microbubbles caused liposome rupture, releasing cargo [156]. This method eliminated the need for any linkage strategies, provided bubble stability, and ensured liposomes and microbubbles were delivered together. These large liposomes were formed at 5 µm in diameter with 1 µm loaded microbubbles and exhibited significantly greater cargo loading efficiency than conventional liposomes. Interestingly, this group encapsulated large protein molecules (IgG, 150 kD) within the liposomes, which can serve as immunotherapeutic agents. Microbubble-loaded liposomes, but not unloaded liposomes, underwent forceful rupture under ultrasound stimulation. Without stimulation, these microcarriers showed stability when incubated in a blood sample [156]. 

Many microbubble–liposome designs have been explored, and they present key advantages including facile fabrication and complexation, expanded cargo loading capacity, enhanced cargo protection, and direct delivery to cells. A key challenge with liposome-based vehicles, however, is that they have lower stability than some other nanoparticle types, and membrane destabilization can result in off-target release of cargo. Liposome stability and lipid layer integrity is controlled by several factors including liposome composition, lipid tail length and saturation, ether linkages, and cholesterol content [124]. Without cholesterol, liposome interactions with proteins can cause membrane instability. Lipid stability and liposome permeability are also dictated by environmental temperature, as the lipid membrane experiences enhanced permeability at the phase transition temperature of the lipids. Instability above the transition temperature leads to liquid disorder and can cause leakage [124,125]. Finally, unmodified liposomes are subject to opsonization, where the particles are tagged with opsonins for clearance by the immune system. Charged liposomes and larger liposomes are frequently opsonized [123]. Particle uptake and clearance by the reticuloendothelial system is mitigated but not entirely eliminated by the addition of PEG stealth coatings, and many liposomal formulations have high accumulation in the liver [157]. Though many design modifications can improve liposome stability, undesired effects, such as lipid oxidation or hydrolysis, membrane destabilization, aggregation, liposome fusion, and immune-based elimination, all still occur [124]. This motivates the implementation of additional classes of nanocarriers to complex with microbubbles for cavitation-based directed delivery.

#### 4.1.2. Microbubble–Polymer Nanoparticle Complexes

##### Polymer Nanoparticles as Nanocarriers

Another class of nanoparticle carrier that has been complexed with microbubbles for ultrasound-controlled delivery is polymer-based nanoparticles, which are illustrated in Table 1. One of the most common and well-characterized polymer materials used for nanoparticle delivery systems is poly(lactic-co-glycolic acid) (PLGA). PLGA is stable in circulation, biocompatible, and biodegradable, resulting in products that are easily metabolized and eliminated by the body [158]. Naturally anionic PLGA nanoparticles can be loaded with hydrophobic or hydrophilic cargo during particle fabrication by using emulsion or double emulsion techniques, respectively. In the emulsion process, polymers and cargo are mixed with water and a surfactant, then sonicated to form nanoparticles [158]. As compared to PEG-coated liposomes, loaded with DOX, PEG-coated DOX-loaded PLGA nanoparticles have shown reduced off-target cardiotoxicity, while preserving anti-tumor therapeutic effects [159]. PLGA nanoparticles have been employed to deliver small hydrophobic drugs, proteins, and nucleic acids; however, loading efficiency, and in some cases, burst release of drugs, remain challenges [158]. 

Another synthetic polymer nanoparticle, the poly(ethylenimine) (PEI) nanocarrier, is hydrophilic and inherently positively-charged, and, as such, it is considered the gold standard for non-viral therapeutic gene delivery from polymer nanoparticles [126,160]. The PEI structure possesses large numbers of protonatable amine groups, which are separated by short alkyl spacers, resulting in a high positive charge density [160,161]. In addition to improving gene complexation efficiency, it is suggested that the positive charge of the PEIs enhances transfection, due to increased cell internalization from electrostatic interactions with cell membranes and endosomal escape, as described below [162].

Natural polymers commonly used in therapeutic delivery, such as chitosan and hyaluronic acid, provide biocompatibility and inherent bioactivity [163,164,165], and complexing naturally-derived polymer nanoparticles with ultrasound microbubbles for ultrasound targeting could be an area of future interest. 

While polymer-based nanoparticle carriers provide some of the same advantages as liposomes, several characteristics of polymers are specifically advantageous to nanocarrier systems, including physical stability in biological environments, ability to confer solubility to otherwise insoluble therapeutics via complexation [127], and diversity in properties and custom design options [126]. Polymer properties vary widely, and cargo loading and delivery abilities are governed by these physicochemical properties. For example, PEI properties are influenced by polymer molecular weight and the extent of polymer branching [162]. While PEI and other cationic polymers, like chitosan, are attractive options for delivery of anionic nucleic acid cargo, PLGA nanoparticles must be modified with cationic molecules when implemented for gene delivery [126,158]. Beyond modifying particle charge for improved cargo loading, multiple polymers are commonly combined in nanoparticle fabrication to optimize properties for specific applications. Adding PEG and poly(caprolactone) to PEI counters toxicity effects, and coating PLGA with chitosan and alginate impacts nanoparticle interaction with the epithelium [126,166,167,168,169,170]. As with other nanocarriers, PEG chains are often added to polymer nanoparticles to reduce immunogenicity and enhance circulation time [121]. Retention of cargo within polymer nanoparticles during circulation is also influenced by the degree of polymer crosslinking, and delivery of cargo is impacted by the type of polymer crosslinking. Noncovalent crosslinking may allow more pH-sensitive, rapid cargo delivery than covalent polymer crosslinking [127,128]. For many PLGA nanocarriers, the mechanism of therapeutic release is hydrolytic polymer degradation-mediated diffusion of cargo, which is tuned by altering the molar ratio of lactic acid to glycolic acid [171].

Many polymer nanoparticle systems have been engineered for targeted drug delivery. Several designs present either polymer–polymer crosslinks or polymer–cargo linkages that are labile to environmental stimuli, such as disulfide bonds (glutathione-sensitive), ester linkages (ROS-labile and esterase-labile), or pH-labile peptide linkers [127,129]. These stimuli are associated with pathological tissue environments and can facilitate degradation of polymer linkages and release of entrapped or linkage-attached cargo. Another polymer-specific method of delivery is network swelling to release the drug from expanded pores. Swelling may occur in response to an endogenous stimulus, such as pH or temperature, which also adds a layer of targeting for controlled cargo delivery [128,130]. Stimulus-responsive swelling behavior is also a critical asset of polymer molecules that enhances direct delivery in cells. Upon endosomal internalization of nanoparticles by cells, many polymer-based nanoparticles undergo pH-responsive swelling, or pH buffering, in response to the acidic endosomal environment, resulting in endosomal swelling and rupture, and, thereby, releasing the nanoparticles directly into the cell cytoplasm [109,110,111,128]. This mechanism is suggested to contribute to improved gene transfection from PEI nanocarriers [111,162].

Polymer nanoparticles are also engineered for active targeting by functionalizing these carriers with biochemical compounds, such as γ-PGA and RGD, for active targeting of tumor-specific or tumor-upregulated markers [127,169]. For remotely-controlled on-demand delivery, polymer nanoparticles have been designed to release their cargo in response to light exposure or changes in temperature [13,127,172,173]. While many existing sophisticated polymer nanoparticles are designed to achieve localized drug delivery through targeting or environmentally-responsive strategies, they are not inherently responsive to ultrasound stimulation. Thus, to confer the design advantages of ultrasound-mediated therapy to different polymer nanoparticles, the nanoparticles can be complexed with microbubbles.

##### Microbubble–Polymer Nanoparticle Complexes for Cargo Delivery

To impart on-demand delivery of DOX from PLGA nanoparticles, DOX-loaded microbubble–PLGA–nanoparticle complexes were developed. PLGA nanoparticles were fabricated and loaded with DOX by the double emulsion–solvent evaporation technique. PLGA polymer COOH end groups were then covalently linked to free-amine-functionalized, perfluoropropane-filled, microbubbles via amide bonds. When implemented in a rabbit liver tumor model, delivery of DOX from this complex, via ultrasound stimulation, resulted in inhibition of tumor growth, improved survival, and, importantly, limited off-target effects [174]. In a different study further investigating this same ultrasound-responsive complex, delivery of DOX-loaded PLGA nanoparticles to rabbit tumor models reduced tumor growth, increasing apoptosis of tumor cells via increased Bax expression and decreased Bcl-2 expression [175]. The double emulsion–solvent evaporation technique of loading DOX into the nanoparticles by encapsulating within the polymer structure during nanoparticle formation was very efficient in both of the above cases, with >85% encapsulation efficiency [174,175]. 

PEI-based nanoparticles for nucleic acid delivery have also been coupled to microbubbles for ultrasound-controlled delivery. One delivery complex incorporating PEI was designed for multileveled targeting of ovarian cancer stem cells, which are an appropriate candidate cell type for on-demand targeted delivery because they are not fast replicating and are. Thus. not as responsive to systemically administered therapies designed for highly proliferative cell populations. In this design, PEI–PEG-based nanocarriers were fabricated with disulfide bonds linking the two polymer types together. Polymer nanoparticles were loaded with plasmid DNA, via electrostatic complexation, and functionalized with biotin for conjugation to perfluoropropane gas-filled microbubbles via biotin–avidin–biotin linkage [176]. Conjugating PEG to positively-charged PEI via disulfide bonds provides an environmentally-sensitive steric shield, which protects the charged nanoparticle from immune responses in circulation, but is cleaved in glutathione-rich and acidic environments, such as tumors, exposing the cationic nanoparticle for cell uptake. This temporary stealth coating imparts tumor-specific targeted delivery. Ultrasound delivery of these particles enhanced the delivery of the DNA payload to the targeted tissues while reducing off-target delivery [176,177]. 

Ultrasound-mediated delivery of GFP plasmid (pGFP) from PEI–PEG nanocarriers to ovarian cancer stem cells in vitro resulted in significantly greater transfection efficiency than cationic commercial transfection agent lipofectamine. When evaluated in vivo, this delivery method also resulted in enhanced transfection efficiency, which was confined to the targeted area, and caused less off-target effects, as compared to lipofectamine treatment in a mouse ovarian tumor model [176]. Upon application of this system for therapeutic delivery of antitumorigenic genes (short hairpin RNA for aldehyde dehydrogenase 1) to ovarian cancer stem cells in vitro, cells showed higher rates of apoptosis than those treated with lipofectamine. Mechanisms contributing to efficacy included enhanced endocytosis and endosomal escape of the plasmids delivered by ultrasound-stimulated PEG–PEI nanocarriers [178]. Future directions for this model include evaluation of therapeutic efficacy in vivo.

PEG has been shown to consistently confer biocompatibility and increased circulation time to nanoparticles by forming a neutral, hydrophilic shield from opsonization [179]. When implemented instead as the base of a polymer nanocarrier, PEG can be modified to optimize other properties for cargo loading and delivery. To form a carrier for delivering sodium borocaptate (BSH), for targeted boron neutron capture therapy of glioblastoma multiforme, PEG was complexed with the polymer segment poly(chloromethylstyrene) (PCMS) to form PEG-b-PCMS. Anionic drug BSH linked to the chloromethyl groups of the PCMS to form the BSH-loaded polymer nanoparticle–drug complex PEG-b-poly((closododecaboranyl)thiomethylstyrene) (PEG-b-PMBSH). PEG-b-PMBSH nanoparticles were electrostatically complexed with cationic microbubbles for ultrasound-mediated BBB penetration and on-demand tumor targeting. This system was delivered in a mouse cranial glioblastoma model, where ultrasound-induced microbubble cavitation caused BBB permeation and rapid boron accumulation in the tumor tissue [180]. This study demonstrated efficient targeted delivery and motivates future work in evaluating therapeutic efficacy of this complex for clinical translation.

One microbubble–polymer nanoparticle complex combining PLGA and PEI was designed to treat renal interstitial fibrosis. PLGA nanoparticles were simultaneously formed and loaded with therapeutic rosiglitazone (RSG, which is an agonist for the anti-inflammatory and anti-fibrotic transcription factor PPARγ) via a double emulsion preparation before complexing with PEI (also via electrostatic linking). As RSG has poor water solubility, PLGA was an apt choice for forming a water-soluble prodrug for effective delivery [181]. The blended polymer nanoparticles presented sufficient positive charge for electrostatic conjugation to commercially available sulfur hexafluoride-filled anionic SonoVue^®^ microbubbles to impart ultrasound responsiveness. This ultrasound-sensitive complex is particularly promising for kidney-related diseases, because ultrasound microbubble destruction has been shown to safely enable renal interstitial capillary permeation for therapeutic delivery. As expected, ultrasound sonication in vitro enhanced nanoparticle uptake in mouse SV40-MES-13 cells. In a rat unilateral ureteral obstruction (UUO) model, intravenous therapeutic complex delivery via ultrasound stimulation effectively reversed PPARγ downregulation, and profibrotic TGFβ, αSMA, and Collagen I expression enhancement caused by UUO. Neither micro-complexes without ultrasound nor therapeutic nanoparticles with ultrasound but no microbubbles were as effective as the microbubble–nanoparticle ultrasound condition, which also attenuated fibrotic collagen deposition [181].

Polymer nanoparticles present many advantages in drug delivery applications and have been successfully utilized in ultrasound-controlled targeted delivery. Development toward effective delivery strategies has also motivated design of a new class of nanoparticles made by combining both lipids and polymers to impart the beneficial properties of both material classes to the delivery vehicles.

#### 4.1.3. Microbubble-Lipid-Polymer Hybrid Nanoparticle Complexes

##### Lipid-Polymer–Hybrid Nanoparticles as Nanocarriers

**Figure 4:**
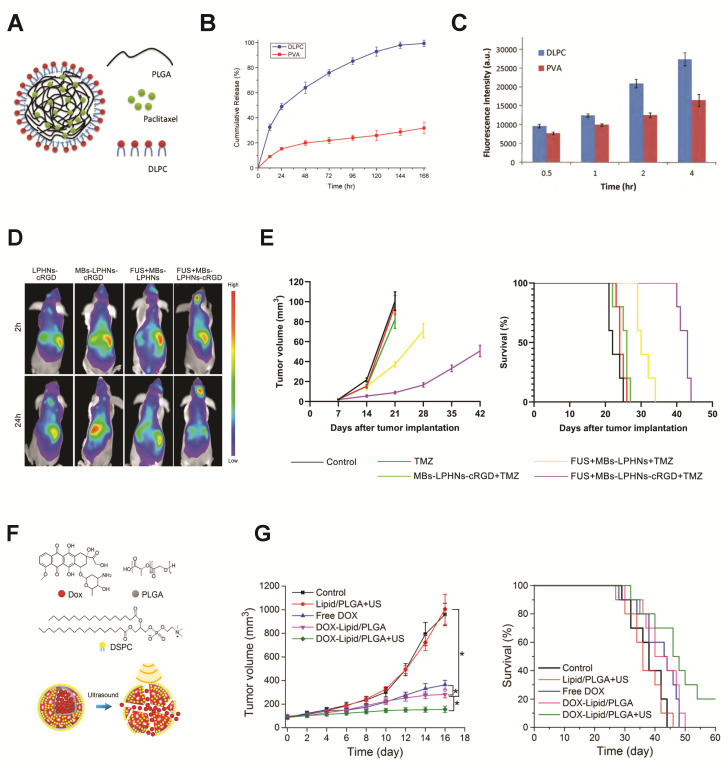
Lipid–polymer hybrid nanoparticles (LPHNs) demonstrate advantages in stability and cargo delivery, and are promising nanocarriers for ultrasound-mediated delivery. (**A**) LPHN composed of a polymer core which houses cargo and a lipid outer layer. (**B**) Cargo release magnitude over time was greater from the LPHNs (DLPC, blue) than from polymer nanoparticles (PVA, red). (**C**) Cargo uptake by cells was greater over time when delivered by LPHNs (DLPC, blue) compared to polymer carriers (PVA, red). (**A**–**C**) reprinted from [133], copyright 2010, with permission from Elsevier. (**D**) Adding ultrasound-responsiveness to LPHNs via microbubble conjugation improved biodistribution and targeting in vivo and enhanced cargo accumulation at the target location in the brain (FUS + MBs-LPHNs-cRGD). (**E**) Using this complex design for the delivery of temozolomide was correlated with inhibition of tumor growth and extended survival (purple). (**D**,**E**) reprinted from [183]: *International Journal of NanoMedicine 2021:16 185–199’* Originally published by and used with permission from Dove Medical Press Ltd. (**F**) Unique model: a microbubble composed of a lipid–polymer hybrid shell, loaded with DOX. (**G**) Ultrasound-stimulated cargo delivery from this system (DOX-Lipid/PLGA + US, green) also resulted in significantly inhibited tumor growth in vivo and greater survival, * *p* < 0.05. (**F**,**G**) Reprinted or adapted with permission from [184]. Copyright 2019 American Chemical Society.

As outlined above, liposomes and polymer-based nanoparticles both have advantageous characteristics resulting from their individual inherent chemical properties. A more recently developed nanocarrier design combines these structural components into lipid–polymer–hybrid nanoparticles (LPHNs), depicted in Table 1. Most commonly organized as a polymer core with a lipid outer layer (Figure 4A), this class of molecule combines the functionality of polymer nanoparticles and lipid-based nanoparticles for multimodal delivery [121,131]. Cargo resides within the inner polymer core, and the lipid layer acts as a barrier to protect and stabilize the core and reduce leakage. An outermost PEG coating may be added to reduce immune clearance of the hybrid particles [121,131]. Like other nanoparticles, LPHNs may be surface-functionalized with moieties for active tissue targeting or for ameliorating the complement activation pathway [131,182]. 

LPHNs are a particularly advantageous class of vehicle for cancer drug delivery. Simultaneous combinatorial drug therapy has shown promise in treating therapeutic-resistant tumors, and LPHNs present several approaches for loading different types of cargo with varying properties, such as hydrophobic and hydrophilic drugs, charged and uncharged molecules, etc., depending upon the nanoparticle fabrication method used. Beyond the multidrug loading capabilities of liposomes alone, these hybrid nanoparticles can be designed to deliver drugs at different timescales or in response to different stimuli [121].

The fabrication process parameters of LPHNs (including lipid to polymer mass ratio and the manner and order in which the molecules are combined) determine the nanoparticles’ physical properties, such as size, loading efficiency, and release kinetics. For example, the outer lipid coating must cover the nanoparticle core completely without gaps to prevent off-target cargo leakage. This coverage, and resulting release kinetics, are determined by the nanoparticle fabrication conditions. As such, a major area of research in the current field of LPHN development for drug delivery is investigating the effects of the preparation and assembly methods on nanoparticle properties and function [121,131,132]. When compared to polymer and lipid-only nanoparticles, drug delivery via LPHNs has shown significantly enhanced sustained drug release and cellular uptake in vitro, and improved delivery to cells in vivo, which indicates their potential for targeted delivery (Figure 4B,C) [121,131,133]. These results are likely attributable to increased sustained drug release and greater carrier stability [133]. Though current studies are limited, localized on-demand delivery of LPHNs can be improved by complexing these nanoparticles with microbubbles to make them ultrasound-responsive. 

##### Microbubble-LPHN Complexes for Cargo Delivery

Brain tumors are difficult to treat due to the protective BBB inhibiting drug delivery. Glioblastoma is resistant to the chemotherapeutic molecule temozolomide (TMZ) by expression of O6-methylguanine-DNA methyltransferase (MGMT), despite TMZ having BBB-penetrative properties. This necessitates delivery of additional therapeutics. A promising approach to increase effectiveness of TMZ treatment is to inhibit MGMT expression via gene therapy, which must be precisely controlled to prevent off-target effects. The high stability of the LPHNs makes them an attractive carrier for this application. Yang et al. elegantly designed a microbubble-LPHN complex with multiple layers of targeting to safely and precisely control delivery of CRISPR/Cas9 plasmids for gene editing to glioblastoma cells in vivo. Here, a PLGA nanoparticle core was complexed with the MGMT-targeting CRISPR/Cas9 plasmids, then combined with biotin- and cRGD-functionalized lipids and self-assembled into LPHNs. The nanoparticles were then bound to perfluoropropane-filled microbubbles by biotin–avidin linking. Encapsulation within the LPHNs protected plasmids from enzymatic degradation and also facilitated sustained plasmid release from the nanoparticles. The functionalization of cRGD served to target the α5β3 surface receptor upregulated in tumor tissue and was correlated with higher T98G glioblastoma cell uptake in vitro. In a T98G orthotopic xenograft model in vivo, focused ultrasound-mediated microbubble cavitation caused BBB opening, and microbubble–LPHN complexes functionalized with cRGD showed significantly greater accumulation in the brain than control LPHN–bubble complexes without active targeting components (Figure 4D). When co-administered with TMZ, ultrasound exposure with microbubble–cRGD–LPHN complexes loaded with Cas9/MGMT plasmids inhibited tumor growth and considerably extended survival period than control conditions TMZ alone, TMZ + cRGD-LPHN–microbubble complexes without ultrasound, and TMZ + LPHN–microbubble complexes with ultrasound (Figure 4E). In this system, each targeting facet enhanced precision of delivery. The ultrasound stimulus-controlled nanoparticle transit across the BBB, the PLGA–core–LPHN facilitated sustained therapeutic release, and the cRGD moiety provided active molecular targeting to tumor cells. Through effective targeted delivery of gene inhibitors against glioblastoma therapy resistance, this technology overcame TMZ resistance to impede tumor growth [183] (Figure 4E). 

Another unique design merged properties of LPHNs and microbubbles into a single carrier. This system combined the echogenicity of lipid microbubbles with the stability and cargo-loading capabilities of polymers. The complex was made via a water/oil/water double emulsion process with ammonium bicarbonate, which decomposed to form the air-filled gas core. The final bubble diameter was 800 nm, with a 30–60 nm thick lipid-PLGA shell. These microbubbles showed greater echogenicity than PLGA-only microbubbles, and were shown to be stable at acidic pH but to undergo cavitation under ultrasound exposure. DOX was loaded into the carriers by dissolving it in ammonium bicarbonate before microbubble formation (Figure 4F). Ultrasound stimulation of the microbubbles facilitated controlled DOX delivery, causing reduced 4T1 tumor cell viability in vitro and targeted accumulation, tumor growth inhibition, and longer survival in a 4T1 tumor model in vivo, indicating the efficacy of this system (Figure 4G) [184]. 

While few studies currently exist that have complexed LPHNs with microbubbles for ultrasound-mediated cargo delivery, the therapeutic outcomes of the above work indicate that this technology is promising and the field is open for more complex LPHN-based delivery designs. 

#### 4.1.4. Microbubble–Protein Nanoparticle Complexes

##### Protein Nanoparticles as Nanocarriers

Another type of nanoparticle implemented in ultrasound-responsive complexes is the protein-based nanoparticle, illustrated in Table 1. As biologically-derived molecules, albumin nanoparticles have inherent bioactivity which contributes to targeting capabilities. Other biologically-derived protein nanoparticles do exist, including gelatin, elastin, gliadin, silk sericin, and others. However, albumin is the most abundant plasma protein, and, thus, it is widely employed for drug delivery, and albumin-based prodrugs are in clinical study [134,135]. Protein nanoparticles are fabricated by emulsion techniques, or, more commonly, desolvation, where the proteins are precipitated out of an organic solution to form nanoprecipitate particles. The addition of glutaraldehyde after precipitation crosslinks albumin molecules together, providing stability to the particles. Fabrication parameters, such as the amount of desolvation solution, pH, and crosslinker concentration, all influence the final albumin nanoparticle size [185,186]. 

Like many other nanoparticle carriers, albumin nanoparticles are 50–200 nm in diameter, facilitating extravasation into tumor tissue via the EPR effect, and they can be functionalized for targeted delivery [186]. However, as natural biological molecules with protein structure, they differ significantly in other ways. Similar to many polymers, proteins are biocompatible and biodegradable, and elicit a limited immune reaction. Unlike most synthetic nanomaterials, however, albumin’s surface innately presents multiple functional groups, including thiol, amino, and carboxyl groups [134]. This intrinsic property is advantageous for linking the nanoparticles to microbubbles or active targeting moieties. The albumin surface site cysteine-34 binds drugs and prodrugs, and free fatty acid binding sites also exist on the surface [135]. The amphiphilic structure of albumin also promotes cargo attachment via hydrophobic interactions. Many common therapeutics, including penicillin and benzodiazepines, bind to the surface of albumin. While active targeting moieties, such as peptides, antibodies, and aptamers, may be conjugated to these surface groups, albumin itself has high binding affinity for tumor endothelial cell surface receptor gp60, resulting in internalization of the nanoparticle [136]. Albumin also binds to several other surface receptors highly expressed in tumor and inflammatory environments, including the glioblastoma-expressed protein SPARC (secreted protein acidic and rich in cysteine) [137,138,139]. These inherent binding capabilities promote precision delivery and accumulation of albumin-based carriers.

Albumin nanoparticles are stable at a range of pH conditions (pH 4–9) and temperatures up to 60 °C, and also have a prolonged circulation time, which is critical for effective EPR-mediated therapy. Albumin has also been shown to accumulate in areas of inflammation, supporting its versatility in therapeutic potential for different pathologies [135]. Albumin nanoparticles can form prodrugs by binding directly to the cargo to impart greater solubility and to extend circulation time. This complexation is particularly effective for short therapeutic peptides with short circulation times [134,136]. While native albumin is nonimmunogenic, modified albumin may trigger immune responses and can be coated with a stealth PEG layer for improved circulation and biocompatibility [137]. One potential challenge of utilizing albumin nanoparticles is aggregation of albumin-covered microbubble complexes due to amine–NHS interactions between adjacent particles. This issue has been ameliorated by adjusting the concentration of drug loaded onto the surface of the nanoparticles to sterically block these interactions [187].

##### Microbubble–Protein–Nanoparticle Complexes for Cargo Delivery

In addition to their advantages for active targeting, the functional groups present on the protein nanoparticle surface are an excellent asset for linking the particles to microbubbles to create ultrasound-controlled delivery systems. A common design scheme consists of covalently linking NHS-functionalized microbubbles to free amine groups on the albumin surface, forming a strong bond.

For enhanced BBB permeation and delivery, NHS-functionalized, sulfur hexafluoride gas-filled lipid microbubbles were covalently linked to albumin nanoparticles by NHS–amine amide bond formation. To evaluate albumin complex delivery, NHS-functionalized Cy5.5 dye molecules were also bonded to the albumin particles. These microbubble–albumin complexes were used to evaluate ultrasound stimulation parameters for safe BBB permeation and delivery of the particles. At optimized settings, ultrasound stimulation resulted in 1.5× greater nanoparticle accumulation in the brain than that of complexes administered without ultrasound [188]. 

Another approach for cargo loading of therapeutic microbubble–albumin complexes involved linking DOX to the albumin nanoparticle surface via electrostatic interactions, resulting in sustained release of DOX from the nanoparticles over an extended time period. This loading strategy was pH-dependent with higher release profiles at acidic pH, which may add another targeting capability to nanoparticles for greater cargo release in tumor environments. Sulfur hexafluoride gas-filled NHS-microbubbles were again linked to the albumin surface via covalent amide bonds, and it was demonstrated that addition of the albumin nanoparticles did not adversely affect echogenicity of the microbubbles. In a VX2 liver tumor rabbit model, more tumor growth inhibition was observed with ultrasound-controlled delivery of these therapeutic particles, using both intra-vascular and more invasive intra-arterial injections, than with intra-arterial delivery of free DOX with ultrasound [187]. 

While several designs have loaded DOX via electrostatic interactions, one group instead thiolated the nanoparticles during desolvation fabrication for DOX attachment. The nanoparticles were then linked to NHS-functionalized perfluoropropane-filled microbubbles via amide linkages. The complexes were dispersed in Lipiodol (oil phase) to form an emulsion. This emulsion was then delivered to a VX2 rabbit liver carcinoma model, serving as an improved ultrasound-targeted delivery method for trans-arterial chemoembolization (TACE) compared to a conventional TACE formulation of DOX in Lipiodol. This ultrasound-based delivery had a greater inhibitory effect on tumor growth than traditional therapeutic methods [189]. 

#### 4.1.5. Microbubble–Metallic–Nanoparticle Complexes and Inorganic Nanoparticle Complexes

##### Metallic and Inorganic Nanoparticles in Therapeutic Delivery

One class of nanoparticles that is in early stages of complexation with microbubbles for delivery is inorganic and metallic nanoparticles. This class, illustrated in Table 1, includes gold, iron oxide, and silica-based nanoparticles, among others, and, like polymer nanoparticles, they can be designed with a range of properties for specific applications. In addition to drug delivery purposes, these particles themselves can serve as therapeutic, theranostic, or targeting agents. Some metallic nanoparticles are inherently responsive to external stimuli. Gold nanoparticles (AuNPs) are responsive to light stimulation and iron oxide nanoparticle accumulation can be directed by magnetic fields [105]. Metallic nanoparticles are favorable drug delivery agents because they are stable and can be easily functionalized for biochemical interactions [140]. While effective, inorganic nanoparticles present some toxicity and solubility issues, making them excellent candidates for microbubble complexation for ultrasound-targeted delivery [105]. Current systems combining metallic nanoparticles with microbubbles for delivery vary widely in both design and application.

##### Microbubble–Metallic–Nanoparticle Complexes and Inorganic Nanoparticle Complexes for Therapeutic Targeting

A few systems exist in which AuNPs are delivered in combination with microbubbles, though many do not complex these components together into a single microparticle, instead administering the nanoparticles and microbubbles independently. In each of these approaches, ultrasound-mediated microbubble cavitation has promoted delivery of nanoparticles to specified regions. An example of this is copper-alloyed-gold nanocluster delivery to the brainstem. The nanoclusters were delivered intranasally and the microbubbles were administered systemically. Focused ultrasound was applied to the brainstem, resulting in enhanced cluster accumulation in the targeted region [190]. In another study, microbubble cavitation enhanced chitosan-coated AuNP delivery to the inner ear by inducing permeability of the round window membrane within the ear [140]. Similarly, microbubble-assisted BBB permeation enhanced the passage of silica-coated gold nanorods into the brain [191]. These results all indicate the potential for targeted drug delivery and future enhanced therapeutic efficiency of therapeutic AuNPs. For all of these approaches, delivery may be enhanced with complexation of the AuNPs to the microbubbles for ensured proximity during bubble cavitation. 

In many current systems, inorganic nanoparticles delivered by microbubbles serve solely as imaging contrast agents, particularly in the case of iron oxide nanoparticles [192,193,194,195]. However, some metallic nanoparticle properties allow them to perform therapeutic roles as well. Inorganic nanoparticles have been implemented as delivery devices for antibiotic agents. Gold and silver nanoparticles promote antibiotic molecule interaction with bacterial cell walls, while some inorganic particles themselves bear antibacterial or antimicrobial properties [196,197,198]. Additionally, metallic nanoparticles, particularly AuNP, have unique optical properties and can convert light to heat for targeted thermoablation therapy. Finally, the facile functionalization of metallic nanoparticles, along with their stability, provides the capability to serve as drug delivery vehicles. 

In recent studies where metallic nanoparticles were complexed to microbubbles, the complexation tended to differ from that of other nanoparticle classes. AuNPs have been incorporated into the microbubble shell itself, while mesoporous silica nanoparticles were either incorporated into the shell or encapsulated within the hydrophobic gas core [199,200,201,202,203]. In one recent study, gold nanorods were incorporated within the microbubble shell, and a PEI-therapeutic DNA prodrug was complexed to the shell. Ultrasound stimulation enhanced accumulation of the nanoparticles and gene therapy payloads at the target site in a hepatoma xenograft mouse model. This gene therapy was combined with colocalized laser-induced photothermal ablation therapy, implemented by the stimulated gold nanorods, resulting in a significant reduction in tumor growth [200]. Similarly, AuNPs incorporated into bovine serum albumin-coated microbubble surfaces accumulated within the ultrasound-targeted region of a U-87 MG xenograft mouse model. Laser irradiation of the delivered AuNPs caused targeted photothermal inhibition of tumor growth [201].

Mesoporous silica nanoparticles (MSNs) are promising drug delivery vehicles, due to their porosity and capacity for loading therapeutic genes via hydrophobic interactions. When MSNs were loaded with a reporter plasmid, complexed with PEI, and then incorporated into a microbubble phospholipid layer, they showed enhanced cell uptake and gene transfection, as well as targeted accumulation in a mouse ovarian tumor model, in response to focused ultrasound stimulation [203]. MSNs may also be modified for additional layers of targeting. In one design, MSNs loaded with the chemotherapeutic tanshinone IIA were functionalized with folate to actively target folate receptor-overexpressing cancer cells, then encapsulated within the hydrophobic core of a microbubble for ultrasound delivery. This complex induced cell apoptosis in vitro. When administered in a mouse H22 tumor model under ultrasound exposure, this complex effectively inhibited tumor growth [202]. 

Ultrasound-controlled delivery of microbubble–metallic nanoparticle complexes has shown enhanced targeting and efficacy in tumor treatment, and this technology can be further expanded for targeted antibacterial treatment applications.

### 4.2. Multi-Stimulus-Controlled Cargo Delivery from Microbubble–Nanoparticle Complexes

**Figure 5:**
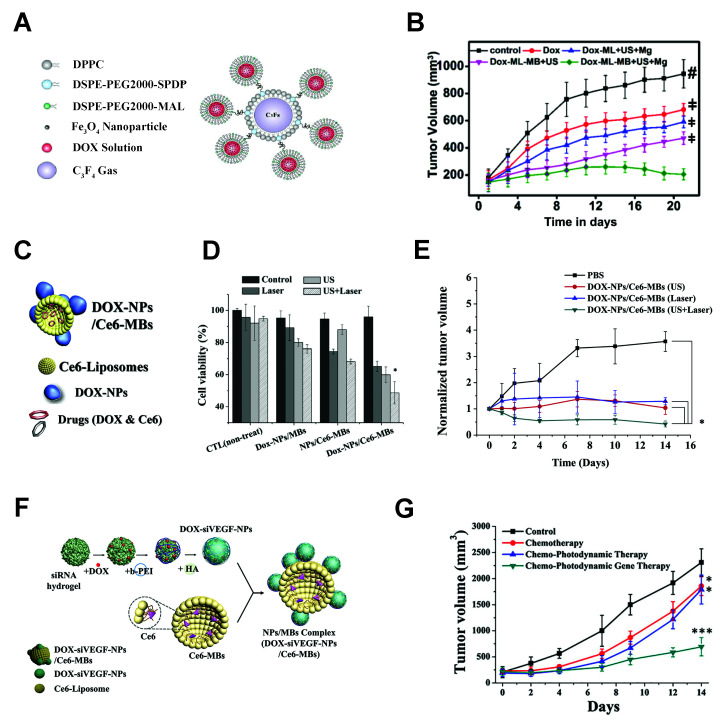
Combined external stimuli enhances cargo delivery from microbubble–nanoparticle complexes. (**A**). Magnetically-responsive iron oxide nanoparticle-containing liposomes were loaded with DOX and covalently linked to microbubbles. (**B**) Dual micro-complex stimulation by both magnetic field and ultrasound (DOX-ML-MB + US + Mg, green) had a greater inhibitory effect on tumor growth than either stimulus alone, ‡ *p* < 0.05, # *p* < 0.001. (**A**,**B**) Adapted with permission from [204], copyright 2020 American Chemical Society. (**C**) DOX-loaded albumin nanoparticles were linked to Ce6 photosensitizer-loaded microbubbles. (**D**) Combining ultrasound-mediated delivery of the DOX and Ce6 photosensitizer with PDT significantly improved treatment efficacy in vitro (DOX-NPs/Ce6-MBs + US + Laser) and (**E**) inhibition of tumor growth in vivo, * *p* < 0.05. (**C**–**E**) reprinted from [206], copyright 2018, with permission from Elsevier. (**F**) Polymer nanoparticles loaded with DOX and therapeutic siRNA were covalently linked to the Ce6-containing microbubbles. (**G**) The combination of chemotherapy, gene therapy, and photodynamic therapy with ultrasound targeting (green line) significantly inhibited tumor growth, * *p* < 0.05, *** *p* < 0.001. (**F**,**G**) reprinted from [207], copyright 2020, with permission from Elsevier.

High precision delivery is particularly important for very aggressive treatment regimens, which can be necessary in cases of highly malignant, accelerated, or late-diagnosed cancers, such as pancreatic cancer. Many of the ultrasound-sensitive complexes discussed thus far have incorporated design components for additional passive, bio-active, or environmentally-stimulated targeting. However, recent approaches have combined multiple external on-demand stimuli for increased precision of targeting with minimal invasiveness. Multi-stimulus microcarriers span multiple classes of nanoparticle components, and they promote precise targeted delivery and high efficacy (Figure 5). 

Towards delivering efficacious therapeutics for pancreatic cancer, a magnetic microbubble–liposome complex was developed to enable magnetic field-influenced accumulation of these therapeutic carriers at a designated location with ultrasound-mediated cavitation to induce targeted delivery. Magnetic liposomes were prepared by rehydrating a lipid thin film layer with an aqueous solution of citric acid-stabilized iron oxide nanoparticles, upon which the magnetic metal particles became housed in the liposome aqueous core. These maleimide-functionalized liposomes were loaded with DOX and covalently conjugated to the thiol-functionalized surfaces of perfluorocarbon-filled microbubbles (Figure 5A). Under ultrasound treatment in vitro, these novel microcarriers caused efficient cellular uptake of DOX by pancreatic tumor cells, resulting in significantly reduced cell viability. In a pancreatic tumor xenograft mouse model, a magnetic field was applied to the tumor region, and tumor growth inhibition was significantly improved when both magnetic and ultrasound stimuli were applied, demonstrating that magnetic-facilitated particle accumulation can enhance efficacy of ultrasound-controlled therapeutic delivery (Figure 5B). These results correlated with the biodistribution of magnetic + ultrasound-controlled delivery of fluorophore as a model drug in healthy mice, where the fluorophore signal was precisely concentrated to one site with magnetic accumulation of liposomes only, but fluorescence intensity was enhanced when microbubbles and ultrasound stimulation were added [204].

Another aggressive form of combinatorial cancer therapy which leverages external stimulation is chemo-photodynamic therapy (PDT). PDT is the chemical excitation of photosensitizer dye molecules by a light stimulus, resulting in local ROS production [205]. ROS cause cell death by oxidative stress, establishing an effective method for killing tumor cells and tumor vasculature. Combining this method with small molecule chemotherapy presents multiple mechanisms of cell death simultaneously for enhanced efficacy. Light stimulation provides spatial control of PDT; however, photosensitizer distribution can be improved by ultrasound-mediated delivery, in part because ultrasound is less scattered by tissue than light.

One elegant system imparted ultrasound targeting to combine photo- and chemical-mediated therapy for improving the safety and efficacy of this already multi-pronged therapeutic approach. The hydrophobic photosensitizer chlorin e6 (Ce6) was loaded onto the tails of the lipid monolayer of perfluoropropane gas-filled, NHS-functionalized microbubbles. To simultaneously load chemotherapeutic agents, human serum albumin (HSA) nanoparticles were formed by disulfide bond crosslinking between thiol groups on albumin molecule surfaces and loaded with DOX. The loaded nanoparticles were then covalently linked to the microbubbles by amine–NHS covalent conjugation (Figure 5C). 

Ultrasound stimulation enhanced cell uptake of both DOX and Ce6 from this complex in vitro, and intracellular Ce6 distribution suggested ultrasound caused delivery through efficient sonoporation, while non-stimulated Ce6 uptake was inferred to be by endocytosis. Laser stimulation of the complex enhanced intracellular ROS production in vitro. When stimuli were combined, cell viability in vitro was significantly decreased (Figure 5D). In a mouse pancreas tumor model, these multitherapeutic complexes inhibited tumor growth significantly more effectively with both ultrasound and laser together than with either stimulus alone (Figure 5E) [206].

While the ultrasound sensitive micro-complex effectively improved chemical–PDT outcomes, potential adverse downstream effects can result from the generation of ROS in PDT. The effective cell death resulting from ROS may cause feedback effects, such as increasing tumor angiogenic signals that enhance growth, requiring an additional form of therapy to combat these effects, often gene therapy to attenuate VEGF upregulation. Further, ROS impede gene therapy efficacy, so delivery of the PDT photosensitizer and genetic material together necessitates a system for both delivery and nucleic acid protection. Current nanocarrier systems incorporating photosensitizers with VEGF inhibitors do not yet successfully undergo targeted delivery, with only 0.7% delivery of administered therapeutic molecules reaching the tumor. As such, the same research group later improved upon their ultrasound-controlled chemo–PDT by complexing the Ce6-loaded microbubble for PDT with a different nanoparticle carrier designed for optimal delivery and protection of genetic therapeutics (siRNA) to combat the unfavorable ROS effects from PDT. 

The perfluoropropane microbubbles were loaded with hydrophobic photosensitizer Ce6 as before, while the therapeutic nanoparticles in this design consisted of siRNA hydrogels loaded with DOX and coated with PEI and HA polymers for protection. To synthesize these particles, anti-VEGF siRNA hydrogel nanoparticles were loaded with DOX before reacting with positively-charged PEI and HA–amine via electrostatic interactions to form a protective coating to prevent RNA denaturation. These amine-presenting nanoparticles were then covalently conjugated to Ce6-loaded, NHS-functionalized microbubbles via an amide linkage (Figure 5F). Physical separation of therapeutic components proved critical, as the siRNA was only effective in blocking the Ce6-induced increase in VEGF expression when delivered from within the separate, polymer-protected nanoparticle.

As before, ultrasound stimulation enhanced breast cancer cell uptake of DOX, Ce6, and siVEGF, and resulted in downregulation of VEGF expression and significantly decreased cell viability in vitro. In a rat cancer model, ultrasound treatment enhanced Ce6 and siRNA accumulation in tumor tissue, and siRNA reduced VEGF upregulation resulting from Ce6 + Laser (PDT) treatment, resulting in greater decreases in tumor growth in comparison to chemotherapy alone or chemo + PDT without siRNA for VEGF reduction (Figure 5G). Importantly, the combined therapy significantly attenuated upregulation of angiogenesis caused by chemo–PDT as evaluated by local blood vessel area. This combined therapy also did not cause major losses in body weight, indicating limited off-target effects. Here, ultrasound stimulation localized microbubble cavitation and rupture of the complexes to just the tumor site, successfully delivering the necessary components for DOX therapy, PDT, and angiogenesis suppression all at the designated location for highly effective therapy [207].

The above multi-stimulus systems show improved targeting and treatment efficacy in comparison to individual stimuli, and their results indicate that combining ultrasound with other on-demand stimuli is a promising future direction for ultrasound-mediated targeted delivery. 

### 4.3. Microbubble–Nanoparticle Complex Linking Strategies

As described above, microbubbles and nanoparticles must be situated in close proximity for microbubble cavitation to cause delivery of nanoparticles to cells, and physically or chemically attaching nanoparticles to the microbubbles ensures this close interaction. As such, microbubble–nanoparticle conjugation is a critical design consideration for ultrasound-controlled nanocarrier delivery, and many linkage types have been employed, as summarized in Table 2. Linkages differ in affinity, and different conjugation strategies are influenced by the inherent properties and chemical groups present on both the microbubbles and nanoparticles themselves.

A commonly-used method for particle linking harnesses the natural non-covalent binding reactions between biotin molecules and protein avidin [122]. This method is straightforward and well established. Both nanoparticles and microbubbles are functionalized with biotin, which does not affect the particle biochemical properties. The biotin groups are then linked together with soluble avidin. Avidin–biotin secondary binding, a naturally occurring reaction utilized for many lab assays, is the strongest non-covalent interaction, though its effectiveness is context-dependent [208]. It should be noted that, due to steric effects from the surface coatings of microbubbles and nanoparticles, a higher soluble avidin concentration is necessary for particle linking than for free biotin binding reactions. Additionally, avidin molecules are charged at biological pH and are, thus, highly repulsive of other avidin molecules, impeding the formation of adjacent biotin–avidin linkages. For improved binding efficiency, avidin may be replaced with neutravidin, a smaller molecule with a lower isoelectric point. Due to the size and lower repulsion between molecules, neutravidin has shown a three-fold higher binding efficiency to biotin than avidin [122,209,210]. One challenge of employing this scheme for linking nanoparticles to microbubbles is that it is a time-consuming process that requires several washing steps, which may result in decreased yield. Additionally, these particles can cause immunogenic responses and some off-target binding, due to endogenous free avidin [149,208]. While employed in some recent complexes, this method was most widely used in early microbubble–nanoparticle complex designs, particularly microbubble-liposomes, perhaps due to the convenience of commercially available lipid–PEG–biotin molecules.

Electrostatic interactions have been implemented to directly link nanoparticles to microbubbles without a biotin–avidin bridge. This scheme is attractive for gene delivery complexes, as anionic nucleic acids may be complexed with cationic nanoparticles, often inherently charged polymers, for delivery. These nanoparticles bind to microbubbles with a negative surface charge (such as commercially available SonoVue^®^ microbubbles) via electrostatic interactions [181]. Alternatively, anionic polymer nanoparticle prodrugs have been efficiently bound to cationic microbubbles, which were synthesized by incorporating cationic lipids into the microbubble lipid membrane. This secondary binding scheme works for microbubble–nanoparticle complexation, but binding stability can depend on physiological conditions. Covalent linkages are the strongest and most robust to environmental factors, making covalent bonding an appealing option for ensuring codelivery of microbubbles and nanoparticles as intact microbubble–nanoparticle complexes.

An early assessment of covalently linked microbubble–liposome complexes evaluated liposome binding efficiency and linkage stability. It was shown that liposomes attach to microbubbles via non-specific chain entanglement; however, significantly greater numbers of liposomes were loaded onto the microbubbles using covalent thiol–maleimide bonding [149]. Thiol–maleimide is an efficient Michael-type reaction that occurs very rapidly [211,212,213]. These covalent linkages were stable when exposed to blood plasma, suggesting their stability in circulation when injected intravenously [149]. When exposed to thiol compounds in biological environments, however, the thiol–maleimide bond may become unstable [213,214]. Similarly, disulfide bonds implemented in microbubble–liposome linkages are stable in circulation but are subject to degradation in reducing environments, which can cause microbubble–nanoparticle separation. In contrast, another “click-chemistry” reaction, used to link liposomes and microbubbles, was the strain-promoted azide–alkyne (SPAAC) DBCO–azide reaction, a relatively rapid reaction that occurs under physiological conditions and does not produce toxic byproducts. Though its reaction rate is slower than that of maleimide–thiol, it may provide greater stability in a range of biological conditions [148]. It can be seen in Table 2 that microbubble–liposome complexes can be bound via a variety of chemistries, as PEG-coated liposomes may be chemically functionalized with different reactive linking groups. Other nanoparticles, such as polymer and lipid–polymer hybrid nanoparticles, may also be modified in order to link to microbubbles. Some nanoparticles, however, inherently present chemical moieties capable of forming covalent bonds. 

Two methods take advantage of existing NHS-reactive functional groups on nanoparticles. PLGA-based nanoparticle polymer chain ends contain carboxylic acid groups, which can be activated to undergo carbodiimide chemistry (COOH + NHS reaction), forming a covalent amide bond with NHS-functionalized microbubbles [208]. Similarly, albumin nanoparticles present primary amine groups, which again can form covalent bonds with NHS-functionalized microbubbles. This method of linkage was employed for all albumin nanoparticle-based complexes surveyed in this review [187,188,189,206,215].

Alternative complex designs implement relative spatial arrangements to combine particles with microbubbles instead of linking their surfaces together. In one design, ultrasound stimulation of microbubbles encapsulated within liposomes resulted in internal bubble fragmentation causing the liposome membrane to rupture and release the housed payload [156]. Polymer nanoparticles that self-arrange into microbubbles can be created through high-speed mixing of gas, nanoparticle solution, and a protein. These nanoparticles can be successfully delivered across the BBB and can effectively deliver drugs to specified sites [216,217,218]. Metallic or inorganic nanoparticles can instead be incorporated within the microbubble lipid layer, and these complexes have also demonstrated effective targeted delivery under ultrasound-induced microbubble cavitation [199,200,201,202,203]. 

**Table 2:** Microbubble–nanoparticle (NP) linking strategies.

Type of Linkage	Schematic	Notable Characteristics	References
Avidin–biotin bridging	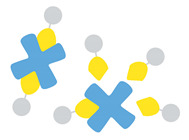	Strong interactions between biotin and avidin, very commonly used, binding efficiency increased with neutravidin. Synthesis is time consuming and results in material waste. Can stimulate immunogenic reactions	Liposomes: [122,146,147,151,153,154] Polymer NP [176,178] LPHN: [183]
Electrostatic bonds	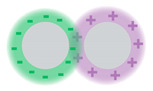	Appropriate for charged nanoparticles, used for cationic polymer nanoparticles for nonviral gene delivery. SonoVue^®^ microbubbles designed for this linking method	Polymer NP: [180,181]
Disulfide bonds	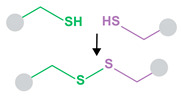	Covalent bonds that can be reversible	Liposomes: [155]
Maleimide–thiol bonds	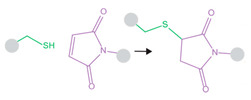	Rapid, biocompatible reaction. Cysteines in proteins have thiol groups on sidechains which may cause non-specific binding, also may cleave at physiological conditions	Liposomes: [155,204,219]
DBCO–azide SPAAC (strain-promoted alkyne–azide cycloaddition)	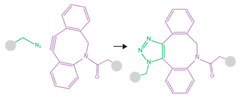	Rapid, nontoxic biorthogonal reaction forms covalent bonds that are stable under biological conditions	Liposomes: [148]
Peptide bonds	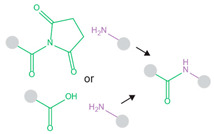	Amide bond formation between Amine and NHS or COOH requires chemical activation, forms stabile covalent linkage. Commonly implemented with albumin nanoparticles which possess surface amines	NHS: Protein NPs: [187,188,189,206,215] NHS: Polymer NP: [207] COOH: Polymer NP: [174,175]
Other complexation strategies	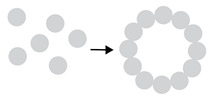	Multiple nanoparticles can be arranged around a gas compartment or embedded in the microbubble shell	Polymer NP: [216,217,218] Metallic/Inorganic NP: [199,200,201,202,203]

## 5. Conclusions and Future Perspectives

Due to limited efficacy of systemic drug delivery and potentially harmful off-target effects of various therapeutics, methods to target drug delivery vehicles directly to diseased tissues remain a clinically-relevant area of active development. On-demand external stimulation of cargo release provides advantageous spatiotemporal control over drug delivery. Ultrasound-stimulated targeted delivery can facilitate controlled delivery through use of applied pressure waves that cause echogenic microbubble cavitation, resulting in the release of attached nanoparticle payload. This attractive option is biocompatible and noninvasive, and the ultrasound can focus to greater penetration depths in tissues compared to other user-controlled targeting stimuli, such as light [15,16]. Ultrasound-mediated drug delivery is enhanced when ultrasound-sensitive bubbles and drug nanocarriers are combined into one complex, and many material options are available for both components. 

In this article, we reviewed current designs for microbubble–nanoparticle complexes, as well as the different schemes for linking particles and microbubbles together. In designing these complexes, the class of nanoparticle used may be selected for particular applications, including fragile or highly toxic cargo, hydrophobicity or charge of cargo, or incorporation of other targeting design elements. Though variation exists within classes, properties relate to nanoparticle type. Liposomes boast facile fabrication and the ability to house both hydrophilic and hydrophobic cargo, but are less stable than other carrier types. Polymer-based nanoparticles are robust in circulation and allow customization. Lipid–polymer hybrid nanoparticles combine the advantages of both molecule types to provide protection and stability to cargo and can deliver multiple cargo types with different properties simultaneously or at different timescales. Protein nanoparticles are stable across a broad pH and temperature range and inherently present functional groups for loading cargo, linking to microbubbles, and for bioactive targeting. Metallic nanoparticles are stable and provide additional targeting layers for thermal ablation therapy or magnetic accumulation. 

Like nanoparticle types and properties, microbubble–nanoparticle linkage strategies vary in technical complexity, biocompatibility, and strength. The linkage strategy depends on the application and inherent properties of each component, though both microbubbles and nanoparticles may be modified with different functional groups for specific linkages. Finally, a few systems have successfully combined the nanocarriers and ultrasound sensitive species in unique ways, such as nesting bubbles within nanocarriers [156], or forming microbubbles from assembled spheres of nanoparticles [216,217,218].

Across different complexation designs, nanoparticle classes, and linkages, ultrasound exposure consistently enhanced targeted delivery via increased in vitro cell uptake of cargo. These results may be largely attributed to local sonoporation, transient formation of pores in cell membranes caused by microbubble cavitation that facilitates direct cellular uptake of released cargo. Ultrasound stimulation was also correlated to improved biodistribution in animal models, showing accumulation of cargo at targeted locations and diminished off-target release and side effects. This was frequently linked to improved therapeutic functional outcomes. Other targeting components, such as nanoparticle size, active targeting moieties, pH-responsive elements, and additional external stimuli, further increased the targeting efficiency of ultrasound-responsive drug delivery complexes. These results are highly promising, and there are many avenues for further advancement of this technology.

As supported by the results described in Section 4.2, a potential future direction for optimized targeting of ultrasound-responsive nanocarrier complexes is the combination of ultrasound with other targeting elements for synergistic targeting precision. Adding ultrasound responsiveness to nanoparticles designed for passive or active targeting has shown improved spatial targeting and efficacy across many studies, and more recent experiments combining ultrasound with light or magnetic external stimuli further improved delivery efficiency and therapeutic efficacy [204,206,207]. Additional combinations of targeting elements, particularly user-controlled stimuli, can enhance precision of targeting, improving therapeutic efficacy [129]. Another future prospect is to expand the range of different nanoparticle types that can be complexed with microbubbles, as well as the amount of cargo that can be carried by these complexes. New nanoparticles, such as supramolecular structures, may improve cargo loading efficiency, leading to greater therapeutic effects, and expand the types of therapy that can be administered with targeted ultrasound [220,221]. Metallic nanoparticles, comprised of gold or iron oxide, have been incorporated within microbubbles to enhance imaging contrast [192,193,194,195]. Direct delivery of metallic nanoparticles by ultrasound from microbubble–nanoparticle complexes for targeted therapy is in early development [199]. These nanoparticles are excellent candidates for ultrasound-targeted delivery, due to presenting solubility and in vivo toxicity complications as free particles [105]. Microbubble–nanoparticle complexes may also be implemented to deliver therapeutic gases to targeted tissues, or to deliver other molecules to specific tissue environments to alter the local extracellular matrix via crosslinking or degradation, rather than targeting resident cells directly. The results outlined in this review highlight the potential of this targeting strategy and motivate future work in translational applications towards clinical trials and clinical implementation. Currently, microbubbles are clinically approved for use as contrast agents [222,223] and some nanoparticles (liposomes, PLGA nanoparticles) have been implemented in the clinic [120,224]. A few studies have tested microbubbles co-delivered with free nanoparticles or free drug for cargo delivery in large animal studies and clinical trials, finding limited adverse effects and some therapeutic efficacy [225,226,227,228,229,230], but linked microbubble–nanoparticle complexes have not yet been widely tested as drug carriers in large animal models or clinical trials. In vitro results suggest that complexed microbubble–nanoparticle platforms can enhance therapeutic delivery efficacy over component co-delivery [117,118,119]. A small number of large animal studies of liposome–microbubble complexes show effective circulation, but therapeutic efficacy was not evaluated [231]. Thus, long term future work consists of moving ultrasound-responsive microbubble–nanoparticle complexes into additional large animal biosafety and efficacy studies before use in clinical trials. One consideration that should be noted for clinical implementation is the limited circulation time for the microbubble–nanoparticle complexes, due to their micron-scale size, which is in the order of <10 min and affects the window during which the complexes can be activated [232,233]. Activation of these particles necessitates training of skilled ultrasound operators to successfully aim and precisely stimulate the targeted tissue. Tailored protocols with adjusted ultrasound parameters must be developed for ultrasound stimulation for this specific application [234]. A major economic feasibility advantage of these drug delivery systems is that they use ultrasound technology and equipment that is already present in many clinics. Overall, ultrasound-directed drug delivery by microbubble–nanoparticle complexes is a valuable advancing technology that has the potential to enhance therapeutic efficacy and improve patient clinical outcomes.

## Figures and Tables

**Table 1:** Nanoparticles implemented in microbubble–nanoparticle complexes.

Type of Nanoparticle	Schematic	Notable Characteristics
Liposome	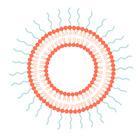	Support endocytosis of cargo [120,121,122], can house multiple cargo types simultaneously [123], stability influenced by environmental factors [124,125]
Polymer	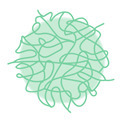	Customizable properties [126], designed for enhanced stability [127], controlled cargo release [126,127,128,129,130], endosomal escape [109,110,111,128]
Lipid–polymer hybrid	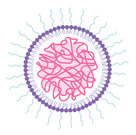	Can house multiple classes of cargo at once and deliver cargos on different timescales [121,131], high stability, due to multilayer design, promotes sustained drug release and cellular uptake [121,132,133]
Protein	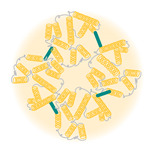	Biologically-derived nanoparticles, inherent biofunctional groups allow for complexing with microbubbles, loading therapeutics, or targeting receptors [134,135,136,137,138,139]
Metallic/Inorganic	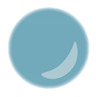	High stability, facile functionalization [140], can be light-/magnetic-responsive [105], can have some toxicity issues [105]
